# The efficacy of elastomeric patient-control module when connected to a balloon pump for postoperative epidural analgesia: A randomized, noninferiority trial

**DOI:** 10.1097/MD.0000000000005828

**Published:** 2017-01-13

**Authors:** Myung Hwa Kim, Yon Hee Shim, Min-Soo Kim, Yang-Sik Shin, Hyun Joo Lee, Jeong Soo Lee

**Affiliations:** a Department of Anesthesiology and Pain Medicine, Anesthesia and Pain Research Institute, Severance Hospital; b Department of Anesthesiology and Pain Medicine, Anesthesia and Pain Research Institute, Gangnam Severance Hospital, Yonsei University College of Medicine, Seoul; c Department of Anesthesiology and Pain Medicine, CHA Bundang Medical Centre, CHA University, Seongnam, Republic of Korea.

**Keywords:** epidural analgesia, patient-controlled analgesia, postoperative pain

## Abstract

When considering the principles of a pain control strategy by patients, reliable administration of additional bolus doses is important for providing the adequate analgesia and improving patient satisfaction. We compared the efficacy of elastomeric patient-control module (PCM) with conventional PCM providing epidural analgesia postoperatively.

A noninferiority comparison was used. Eighty-six patients scheduled for open upper abdominal surgery were randomized to use either an elastomeric or conventional PCM connected to balloon pump. After successful epidural catheter insertion at T_6–8_ level, fentanyl (15–20 μg/kg) in 0.3% ropivacaine 100 mL was administered at basal rate 2 mL/h with bolus 2 mL and lock-out time 15 minutes. The primary outcome was the verbal numerical rating score for pain.

The 95% confidence intervals for differences in pain scores during the first 48 hours postoperatively were <1, indicating noninferiority of the elastomeric PCM. The duration of pump reservoir exhaustion was shorter for the elastomeric PCM (mean [SD], 33 hours [8 hours] vs 40 hours [8 hours], *P* = 0.0003). There were no differences in the frequency of PCM use, additional analgesics, or adverse events between groups.

The elastomeric PCM was as effective as conventional PCM with and exhibited a similar safety profile.

## 1 Introduction

Patient-control analgesia (PCA) has been the gold standard for postoperative pain management since it was introduced nearly 30 years ago. Disposable mechanically controlled pumps that use an elastomeric reservoir remain widely used,^[1]^ while the operational complexity of electronic pumps raises concerns about potentially introducing dangerous programming errors.^[2,3]^ The simplicity of elastomeric devices makes it as an easy and straightforward way to control postoperative pain, particularly in ambulatory care using local anesthetics.^[4]^

Generally, an elastomeric pump is designed to administer the analgesic in the manner of a continuous, demand-independent background infusion combined with bolus doses on demand.^[5–7]^ When considering optimal pain control strategy for patients undergoing major surgery, timely and reliable additional bolus doses of pain medication when needed are important for patient safety and satisfaction.

**Figure 1:**
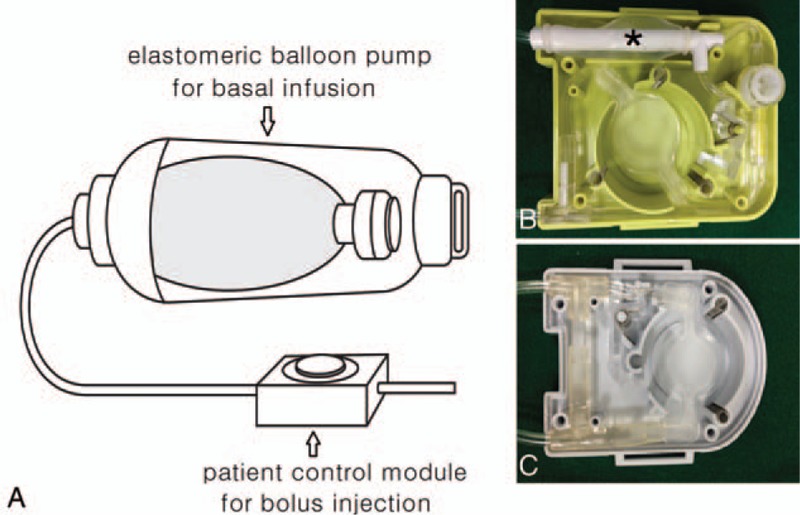
(A) Schematic drawing of a elastomeric balloon patient controlled analgesia. (B and C) Internal structure of elastomeric (B) and conventional (C) patient control module. ∗2nd elastomeric balloon pump.

A patient-control module (PCM) connected to balloon pump is designed to administer a bolus dose by pressing an attached button (Fig. 1A). Conventional PCM consists of a small plastic, nonelastic bag filled with analgesics under the button (Fig. 1C). While the patient presses the button on the conventional PCM, a bolus dose within the small plastic bag is delivered to the patient. However, conventional PCMs barely overcome the resistance during epidural PCA, which requires drug delivery to the relatively resistant epidural space through a long, narrow catheter. The elastomeric PCM has a secondary elastomeric balloon connected to the conventional plastic bag under the bolus button (Fig. 1B). The elastomeric PCM requires a short button press to efficiently deliver the bolus dose, unlike conventional PCMs, which require relatively prolonged and high pressure on the bolus button to achieve the same outcome.

This randomized, noninferiority study compared the efficacy of elastomeric and conventional PCM connected to a balloon pump in providing postoperative epidural analgesia over 48 hours in patients undergoing open upper abdominal surgery.

## 2 Methods

### 2.1 Participants

This randomized, double-centre, open-label, parallel-group, noninferiority trial was approved by the Institutional Review Boards of the 2 study hospitals and registered at ClinicalTrials.gov (ref: NCT 01976494). After providing written informed consent, 86 patients (aged 20–70 years, and American Society of Anesthesiologists class I–II) who were scheduled for an elective open upper-abdominal surgery and postoperative epidural analgesia were enrolled from November 2013 to July 2014 at Shinchon and Gangnam Severance Hospital, Yonsei University Health System (Seoul, Korea). Exclusion criteria were a history of renal or hepatic insufficiency, neurologic diseases, suspected or known allergy to local anesthetics or opioids, bleeding tendency according to clinical or laboratory findings, active infectious disease, infection at the epidural needle puncture site, epidural catheter insertion failure or an inadequate epidural block, and difficulties in communication.

A researcher who did not participate in managing the anesthesia and assessing the data prepared the randomization schedule using a computer-generated sequence. Patients either received a PCA pump with an elastomeric PCM, Elastomeric group (Accufuser Omnibus; Woo Young Medical Co., Ltd, Seoul, Korea) or a conventional PCM, Conventional group (Infusor SV; Baxter Healthcare Corp., Deerfield, IL). Both devices had an identical 2 mL/h basal flow rates, 2 mL bolus dosing, 15-minute lockout time, and 100 mL reservoir volumes.

### 2.2 Anesthesia management

Epidural catheter insertion and anesthesia management were performed by experienced anesthesiologists. All patients received intravenous premedication with 0.05 mg/kg of midazolam and 0.1 mg of glycopyrrolate. After routine monitoring, epidural catheterization was performed using an 18-gauge epidural catheter (Epidural Minipack SYSTEM 2; Smiths Medical, Inc., Ashford, UK), and the catheter tip was placed at the T6–8 level. To confirm accurate catheter placement, 3 mL of 1% lidocaine containing 5 μg/mL of epinephrine was administered via an epidural catheter, and the block level was determined by cold sensation response to an alcohol swab 10 minutes after the injection. If sensory block was not achieved at the target level dermatomes, the epidural catheterization was considered to have failed, and the patient was excluded from the study. After successful epidural catheter insertion, the assigned PCA device was filled with fentanyl (15–20 μg/kg) in 100 mL of 0.3% ropivacaine. Baseline weight of the prepared PCA device was measured using an electronic scale.

After anesthesia was induced with propofol, remifentanil, and rocuronium, it was maintained with an inhalation anesthetic, remifentanil, and rocuronium. When closing the peritoneum, 7 to 10 mL of 1% lidocaine was injected slowly into the epidural catheter, and the prepared PCA equipment was connected to the epidural catheter. All patients received ramosetron (0.3 mg) intravenously before completing the surgery to prevent postoperative nausea and vomiting. At the end of the surgery, all anesthetics were discontinued, and neostigmine and glycopyrrolate were intravenously administered to reverse the neuromuscular block. After confirmation of adequate recovery of spontaneous ventilation and consciousness, patients were extubated and transferred to the postanesthesia care unit (PACU).

### 2.3 Data collection

The day before operation, patients were informed about the verbal numerical rating score (VNRS; 0 = no pain, 10 = worst pain possible) for pain assessment and how to use the PCM, and postoperative visits were scheduled by the investigators. VNRS for pain was assessed at the following periods from PACU admission: 0 to 1, 1 to 3, 3 to 6, 6 to 12, 12 to 18, 18 to 24, and 24 to 48 hours postoperatively. Patients were asked to rate their worst pain during each time period. Also, the frequency of PCM use, change in the VNRS after using the PCM, and frequency of rescue analgesics administrated were recorded. To evaluate the reliability of drug delivery, actual and predicted amounts of the drug administered to the patient were compared twice during the study period (12–18 and 24–48 hours postoperatively, respectively). To evaluate the actual amount of drug administered, the investigator weighed the PCA devices at the patient's bedside using a portable electronic scale. From the weights at the time, we accurately calculated the amount of drug administered. The predicted amount of administered drug was calculated by multiplying the PCM-use events and bolus volume (2 mL), and multiplying the total infusion time and basal flow rate (2 mL/h). Postoperative adverse events, including hypotension, nausea/vomiting, headache, dizziness, sedation, urine retention, and sensory changes were also assessed at each follow-up period.

### 2.4 Statistical analysis

Statistical analysis was performed by a medical statistician who was unaware of the group allocation. SAS software, version 9.2 (SAS Institute, Inc., Cary, NC) was used. The primary endpoint was the overall VNRS for pain. A prior survey at our institution that queried patients receiving epidural analgesia after open gastrectomy showed that the median (interquartile range [IQR]) VNRS assessed within 6 hours postoperatively was 5 (4–6). Given a noninferiority margin of 1, a sample size of 37 patients in each group was estimated to be required to obtain 80% power with an alpha level of 0.05. Considering the dropout rate of 20%, 43 patients per group were recruited and randomized.

Values are mean (SD) or patient number (proportion). Mean differences with 95% confidence intervals (CIs) for VNRS pain between the 2 groups were calculated by the method of Hodges–Lehmann. Data were analyzed using the Chi-square or Fisher exact tests for categorical variables and the independent 2-sample *t* test for continuous variables. Repeated measures data were analyzed using a linear mixed model. Multiple comparisons between the study groups on each time point were adjusted using the Bonferroni correction, and *P* values shown are corrected values. Nominal variables were analyzed using the Chi-square or Fischer exact tests when appropriate. *P* values <0.05 were considered statistically significant.

## 3 Results

**Figure 2:**
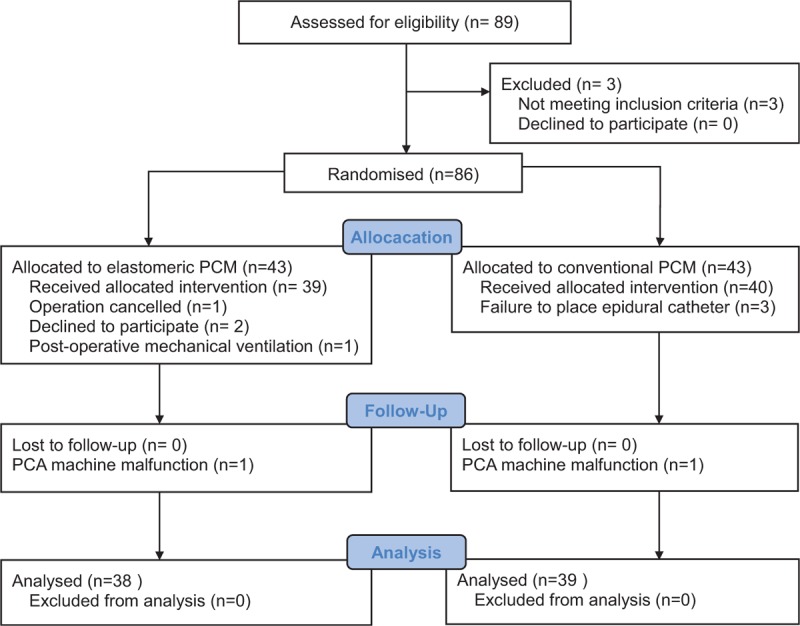
CONSORT flow diagram of the study design and patient selection. PCM = patient-control module.

Of 89 initially enrolled patients, 86 were randomized into the 2 study groups (Fig. 2). After excluding 9 patients, 77 were eligible for analysis. Demographic and surgical characteristics were similar between the 2 groups (Table 1). The total amount of fentanyl used in epidural PCA did not differ between the groups (Table 2).

**Table 1:**
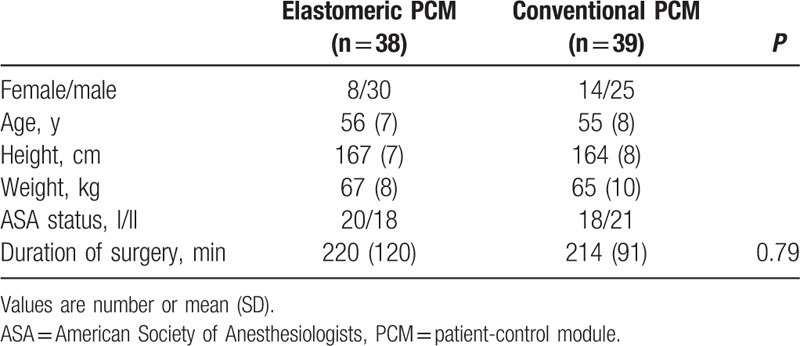
Patients’ and surgical characteristics.

**Table 2:**
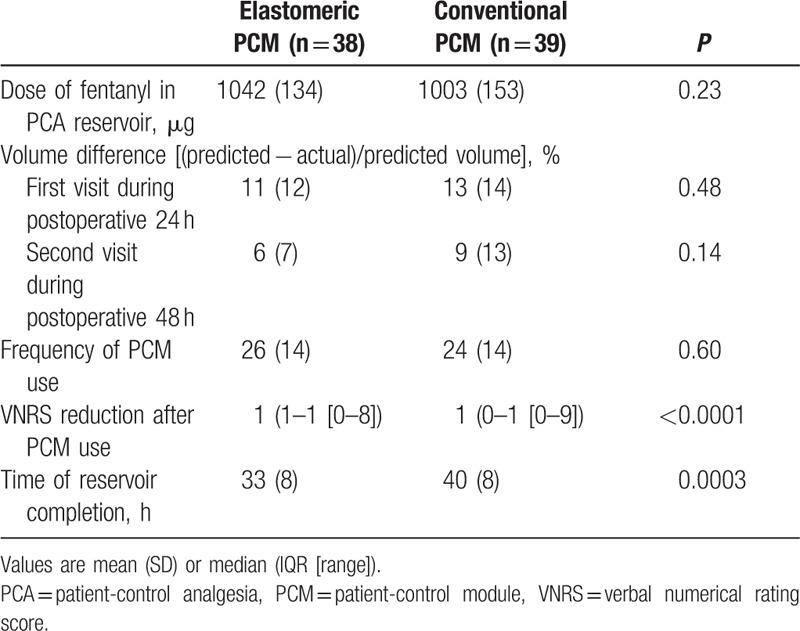
Data related to the patient-control analgesia (PCA) pump.

**Figure 3:**
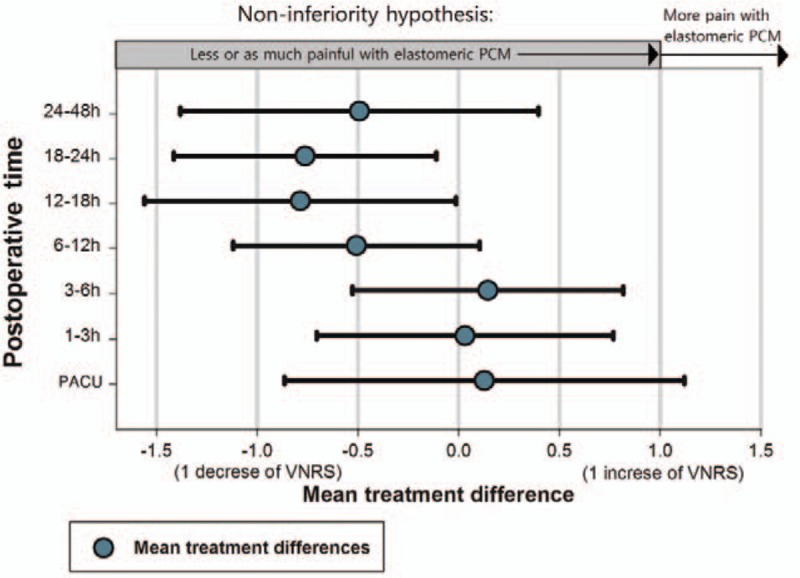
Mean treatment differences regarding the pain score (2-sided 95% confidential interval). The margin of noninferiority is 1.0 for the pain verbal numerical rating score.

**Figure 4:**
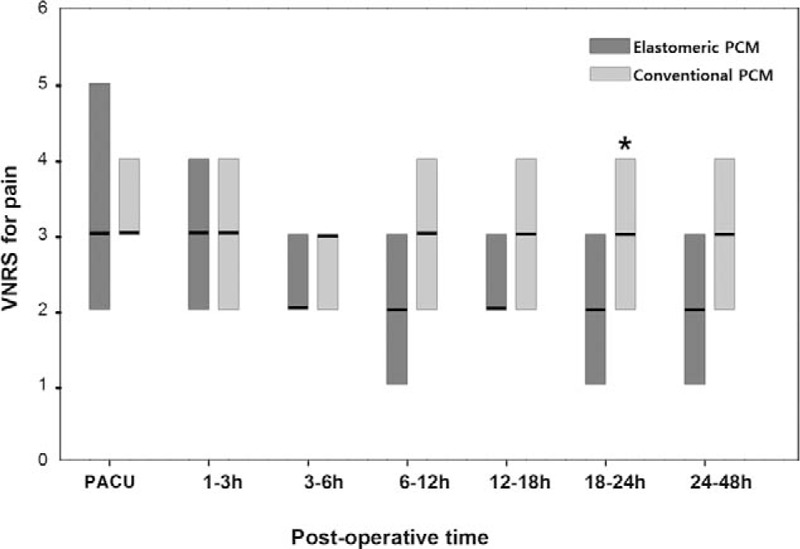
Verbal numerical rating scores for pain during 48 hours postoperatively in patients using elastomeric patient-control module or conventional patient-control module. ∗*P* < 0.05 compared with elastomeric patient-control module (Bonferroni corrected).

The 95% CIs for the mean treatment differences in the pain VNRS during 48 h postoperatively were <1, indicating noninferiority of the elastomeric group, except during time in the PACU (Fig. 3). There were no significant differences in the median (IQR) pain VNRS, except during 18 to 24 hours postoperatively (2 [1–3] in the elastomeric group vs 3 [2–4] in the conventional group; *P* = 0.02) (Fig. 4).

The actual volume infused was less than the predicted volume in both groups at any of the times examined (Table 2). Differences between the predicted and actual volume infused through the PCA pump were similar between the groups. In addition, the frequency of PCM use was similar between the groups. However, the reduction in VNRS for pain after using the PCM was larger in the elastomeric group than the conventional group. Moreover, completion of the PCA reservoir occurred earlier in the elastomeric group than in the conventional group.

There were no differences in the incidence of adverse events between the 2 groups (Table 3). The number of rescue analgesic and antiemetic uses were not different between the groups.

**Table 3:**
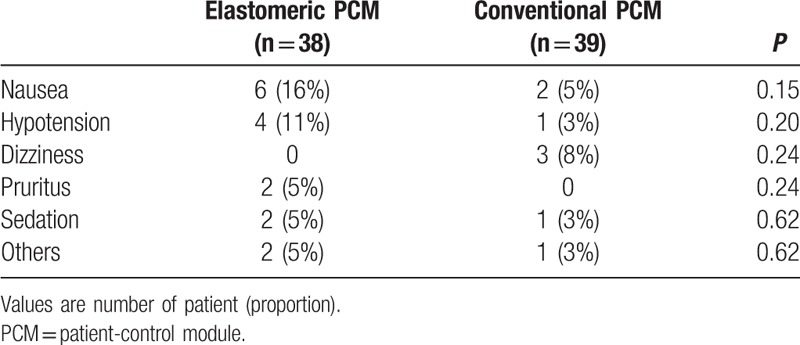
Adverse events.

## 4 Discussion

To the best of our knowledge, we provided the first report on the comparison between elastomeric and conventional PCMs connected to a balloon pump for providing epidural analgesia. This noninferiority comparison demonstrated that the elastomeric PCM was as effective as conventional PCM, with similar safety profiles, in providing epidural analgesia after upper abdominal surgery. In addition, when a pump reservoir was connected to the elastomeric PCM, the dose was completed earlier than with conventional PCM despite the same basal infusion rate and similar frequency of bolus button use. This suggests that the elastomeric PCM may be an effective alternative for the successful delivery of a bolus dose during epidural PCA.

The fundamental concept of PCA is self-administration of analgesics on patient demand. Thus, whether the demand dose is successfully administered to the patient by pushing the button on the PCM to produce appreciable analgesia remains paramount to the efficacy of PCA. From previous research into patient perspectives regarding PCA, 22% believed that uncertainty about PCM made their pain worse. Furthermore, most patients preferred the design of an easy-to-use PCA that is able to deliver more medicine.^[8]^ Thus, with adequate training of patients about the PCM, easy and proper functioning of the PCM bolus injection feature is very important to ensure successful use of PCA.^[4]^

**Figure 5:**
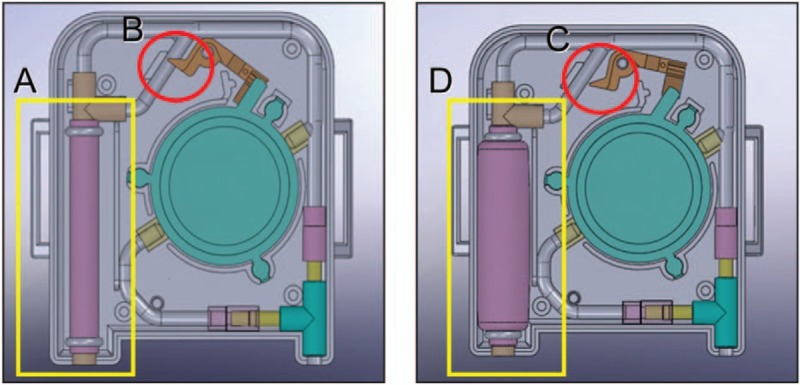
Schematic diagram of the elastomeric patient control module illustrating its operating principle. Inside structure of the module: preexpansion state of the second elastomeric balloon (A) while the crank closes the connection tube (B). When the bolus button is pushed, the crank moves, which allows the connection tube to be opened (C). Pressure on the elastomeric reservoir bag delivers the analgesic to the second elastomeric balloon, resulting in its expansion (D).

Conventional PCMs equipped with a small compressible plastic bag underneath the bolus button can barely overcome the flow resistance of long and narrow gauge epidural catheters. An in vitro test performed by 1 manufacturer (Woo Young Medical Co., Ltd) showed that a 1-mL bolus of medication could be completely delivered in 40 to 80 seconds with the average pressure (7.57–9.48 kgf) a patient exerts when pressing the bolus button of a conventional PCM connected to an epidural catheter. In clinical settings, patients suffering from postoperative pain cannot press the button for such a long time; as a result, the bolus may not be appropriately delivered, and a dose-related error may occur. In addition, applying excessive pressure on the button increases the chance of bursting the bolus bag. However, in the case of elastomeric PCMs, a short push on the button is enough to fill the small elastomeric balloon in the PCM while the crank is open, and the bolus dose is delivered to the patient at a constant speed by the innate elasticity of the second bag. Additionally, the crank in the elastomeric PCM prevents backflow of analgesics (Fig. 5). Theoretically, the use of an elastomeric PCM is less affected by the resistance from the long, narrow catheter of the epidural PCA.

In this noninferiority trial, the elastomeric PCM was as effective as the conventional PCM in providing epidural PCA postoperatively, with a similar safety profile. Epidural PCA with fentanyl and ropivacaine provided adequate analgesia in both groups during the first 48 hours after upper abdominal surgery, in accordance with a previous study that used epidural PCA with opioid and local anesthetic.^[9]^ Even though there were no significant differences in pain VNRS between the groups except at 1 time point (18–24 hours postoperatively), PCA with elastomeric PCM may have provided better pain relief during the study period. In addition to the analgesic efficacy, the safety profile can be another concern in choosing PCA devices. The incidence of PCA device malfunction resulting in under-dosing was 2% in both groups. In the case of opioid use in PCA, fears of overdose and addiction can limit effective management.^[10–12]^ In our setting, opioid-induced adverse events were not severe and were observed in both groups with a similar incidence.

Actual volumes infused through the PCA devices remained within 15% of their predetermined volumes in both groups, in accordance with a previous report regarding the disposable balloon pump.^[13]^ Discrepancies between actual and predetermined volumes did not show any differences between the groups. Despite these results, the reduction in VNRS for pain after using the PCM was larger in the elastomeric group than conventional group. In addition, the PCA balloon pump connected to the elastomeric PCM was completed earlier than with conventional PCM despite the same basal infusion rate and similar frequency of bolus button use. This suggests that the elastomeric PCM improved the administration of the bolus dose compared to conventional PCM in epidural PCA. This suggests that the elastomeric PCM may be an effective alternative for the successful delivery of a bolus dose during epidural PCA. In addition to the reliable administration of a bolus dose, an appropriate feedback loop to inform patients whether they receive the medication should be developed, especially because the perception of pain control increases patients’ satisfaction.^[8]^

There were some limitations in the present study. First, because the PCM apparatuses were different between the 2 groups, the investigators who collected the data could not be blinded to the randomized groups. To minimize possible bias, the same protocol was applied strictly to both groups throughout the study period. Second, we did not survey patients’ satisfaction. Although patient's satisfaction is considered an important outcome in evaluating the efficacy of PCA devices, it is complex; patient satisfaction surveys tend to be positive because patients are unwilling to criticize their treatment,^[10]^ and they may find that the pain is not as bad as expected.^[14]^ A numerical rating scale for pain may reflect patient satisfaction, which correlates with a lower pain intensity.^[15,16]^ Third, pain control tended to be superior in the elastomeric group, although VNRS for pain at only 1 time point (18–24 hours postoperatively) showed statistical significance. The study sample size was calculated for a noninferiority trial, which generally requires a smaller sample size than a superiority trial. Thus, with a sample size of 77, the present study was underpowered to definitively assess the superiority of 1 device with respect to the pain VNRS.

## 5 Conclusion

The findings of our study indicate that an elastomeric PCM, when connected to a balloon pump, is as effective as a conventional PCM for providing epidural analgesia after upper abdominal surgery, without producing significant complications. Furthermore, the elastomeric PCM improved pain control after using the PCM.
