# The efficacy of elastomeric patient-control module when connected to a balloon pump for postoperative epidural analgesia: A randomized, noninferiority trial: Erratum

**DOI:** 10.1097/MD.0000000000006079

**Published:** 2017-01-27

In the article “The efficacy of elastomeric patient-control module when connected to a balloon pump for postoperative epidural analgesia: A randomized, noninferiority trial”,^[1]^ which appeared in Volume 96, Issue 2 of *Medicine*, Dr. Myoung Hwa Kim's name was originally spelled incorrectly.
